# Optimizing cyclopean stimuli for the evaluation of stereo vision by steady-state visual evoked potentials

**DOI:** 10.1007/s10633-025-10059-6

**Published:** 2025-10-17

**Authors:** János Radó, Eszter Mikó–Baráth, Péter Hegyi, Vanda A. Nemes, Gábor Jandó, Péter Buzás

**Affiliations:** Institute of Physiology, Medical School, University of Pécs, Szigeti út 12, Pécs, Baranya, 7624 Hungary

**Keywords:** Cyclopean VEP, Dynamic random dot correlograms, Dynamic random dot stereograms, T^2^_circ_-test, Cortical binocularity

## Abstract

**Purpose:**

This study aimed to optimize dynamic random dot correlogram (DRDC) and stereogram (DRDS) stimuli to evoke steady-state visual evoked potentials (ssVEP) on multiple EEG channels for the objective assessment of stereopsis.

**Methods:**

EEG recordings were conducted on 22 healthy adults (mean age: 30.2 ± 5.8 years) while viewing cyclopean and control stimuli. DRDC and DRDS were presented at three temporal frequencies (0.9375, 1.875, and 3.75 cycles per second, cps) using anaglyphic channel separation. The ssVEP responses were analyzed using T^2^_circ_ statistical test to determine the most effective stimulus for eliciting significant cortical activity.

**Results:**

DRDC at 1.875 cps evoked significant ssVEP responses in 93% of participants on at least one occipital electrode (O1, Oz, O2) and in 100% when including parietal-occipital electrodes. DRDS at similar frequencies also produced robust responses but required additional parietal electrode monitoring. Monocular control measurements confirmed that responses were stereo-specific.

**Conclusions:**

DRDC at 1.875 cps was the most effective stimulus for objective electrophysiological assessment of stereopsis, demonstrating high reliability with minimal electrode setups. These findings support the integration of optimized ssVEP protocols into clinical assessments, particularly for non-verbal or pediatric populations.

**Supplementary Information:**

The online version contains supplementary material available at 10.1007/s10633-025-10059-6.

## Introduction

The goal of the present study was to develop a protocol for visually evoked potentials (VEPs) that can accurately determine the presence of stereopsis in healthy adult participants with normal binocular vision. To achieve this, we used stimuli that selectively activate the neural mechanisms underlying binocular depth perception, also called stereopsis. Based on the scientific literature of several decades [1–3], the most specific stimuli for this purpose are cyclopean pattern, such as dynamic random dot stereograms (DRDS) and correlograms (DRDC). These eliminate monocular form cues and rely on binocular information, using a dynamic field of random dots. DRDS contain true binocular disparity, resulting in a clear and localized sense of depth. DRDC, in contrast, provide depth cues through interocular correlation of dot positions without binocular disparity, leading to a more diffuse depth percept. Despite this difference, both stimuli are widely used in stereopsis research due to their ability to selectively engage disparity-sensitive neural mechanisms [2].

When presented dichoptically (e.g., via anaglyph red-green glasses), these patterns evoke a three-dimensional percept in observers with intact stereopsis, and appear as random flickering dots under monocular viewing. Proper channel separation is therefore essential for effective stimulus delivery. Measuring behavioral or electrophysiological responses to DRDS and DRDC have become the standard when objective evaluation of stereoscopic perception is required and verbal responses are not available or not reliable, such as in animals [4, 5], infants or children [6–8]. A missing response to such stimuli indicates diminished or missing stereopsis that can be due to amblyopia or the presence of amblyogenic conditions [9–11]. Stereopsis can be impaired by abnormal visual experiences due to incoherent retinal images, often caused by monocular blur from refractive errors, strabismus, or unilateral eye disease. Persistent binocular vision disturbances in childhood may lead to amblyopia. Beyond optical and oculomotor factors, stereopsis deficits may also result from neural processing impairments within the visual pathways or cortex. While the lack of response to random dot stereo stimuli primarily detects impaired or absent stereopsis, it may also indicate broader visual developmental abnormalities [12]. An objective electrophysiological marker for stereo vision disturbances would therefore be especially useful for the evaluation of pediatric patients and could be integrated with other VEP protocols.

Various visual depth percepts, such as spatial surfaces or temporal changes in depth can be generated by using DRDS or DRDC [2, 13]. The wide range of possible depth illusions raises the question of which stimuli are most suitable for eliciting objectively measurable electrophysiological responses. It is important to consider that the perceptual salience of the depth encoded features is not necessarily a good indicator of the amplitude or reliability of the evoked electrical responses. Even if a stereogram is clearly visible, it may not evoke proper VEP or trigger enough neuronal activity to surpass statistical thresholds. The reliability of the VEP response can be further compromised when the examinee is moving or inattentive, which is sometimes the case with children.

Circular T-squared (T^2^_circ_) statistics [14] and other bivariate methods [15] are beneficial in the evaluation of steady-state VEPs as they provide a robust approach for detecting and analyzing periodic signals in the presence of noise and naturally occurring background EEG activity. These statistical tests help in distinguishing significant VEP responses and improve the accuracy of diagnosing visual pathway integrity [8, 16].

DRDC and DRDS are still a large family of possible stimuli that must be specified by a range of parameters defining the random dot carrier and the spatial and temporal characteristics of the disparate features responsible for the stereoscopic effect. There is no consensus in scientific literature on the most effective set of parameters that evokes a VEP response. Based on the broader scientific literature (see Table 3) [2, 6, 9, 17, 18], our previous works [7, 19–21], and pilot measurements preceding the current study (see Methods and SI Table 1), two specific stimulus types, incorporating periodic temporal changes at three temporal frequencies, were selected for further investigation.

Here, we sought to identify the stimulus that produces a statistically significant and reproducible response in the largest number of participants with intact stereopsis.

1. Which stimulus type, DRDS or DRDC, elicits the most robust cortical response in VEP measurements?
2. Which temporal frequency (1–4 cycles per second) is optimal for eliciting a reliable response?
3. Can stereopsis be consistently and objectively identified through electrophysiological recordings in individuals with psychophysically confirmed stereopsis?

## Methods

### Participants

In our cross-sectional study, we examined 22 healthy volunteers aged 22–42 years (mean age: 30.2 ± 5.8 years, SD, 68% female) with normal or corrected to normal visual acuity and no history of eye disease and surgery. The study was approved by the Regional Research Ethics Committee at the University of Pécs (registration number: 5638-PTE), and all participants provided both verbal and written informed consent after receiving detailed information about the study.

Visual acuity was assessed at the stimulus viewing distance of 100 cm using a custom-designed acuity chart. The participants' visual acuity was − 0.09 ± 0.07 (SD) logMAR. When needed, participants wore their best-corrected prescription throughout the whole testing process. Each participant underwent the TNO stereo vision test (Lameris Ootech bv, Belgium, 6716 WC Ede, Da Vincilaan 7) following the manufacturer’s instructions, and all demonstrated a stereoacuity of at least 120" (seconds of arc viewing angle). Prior to VEP measurements, we ensured that all participants could perceive the depth-encoded features in the cyclopean stimuli. An additional inclusion criterion was a normal ssVEP response to luminance-defined checkerboard (see *Results*).

### Pilot measurements

In a pilot study, we compared ssVEPs evoked by various DRDC and DRDS stimuli systematically to measure cyclopean vision in healthy adults (Radó et al., International Neuroscience Conference, Pécs 2024, p. 211. P6.25 and Radó et al., ECVP 2024; E-Abstract p. 112, P268). Altogether, 23 participants and 31 measurement sessions were included. This pilot study was an independent measurement series, separate from the main study. We optimized cyclopean stimuli by systematically refining the spatial pattern, spatial and temporal frequency, dot size and refresh rates to identify those that produced the strongest and most reliable ssVEP responses (SI Table 1). When analyzing the 3 occipital electrodes only, the most effective stimuli featured 3.75 cps (cycles per second) DRDC with full-field or checkerboard reversal appearance, a 3.5' (minutes of arc) dot size and 60 Hz refresh rate.

### Stimuli and experimental setup

Stimuli were generated using MATLAB (The MathWorks, Inc., USA) with Psychophysics Toolbox version 3 (PTB-3) extensions [22, 23]. The stimuli were displayed on a CRT monitor (Samsung Syncmaster 957 MB S) with a dot size of 3.5’, and a dot refresh rate of 60 Hz synchronized to the frame refresh of the monitor. The viewing distance was 100 cm, covering a visual field of 1194’ × 818’. A yellow circular fixation point (41’ diameter) was presented at the center of the screen. Measurements were conducted in a darkened room.

The experiment included 1) high-contrast, non-stereo, steady-state checkerboard reversal stimuli and 2) cyclopean stimuli as follows:

First, non-stereo checkerboard reversal with check sizes of 60’ and 15’ were used to evaluate the quality of ssVEP response from each participant. These stimuli were presented according to the ISCEV standard protocol [24], except for the temporal frequency, which was set to 3.75 cps (7.5 reversals per second, rps). This frequency was chosen to match the reversal rate of the stereo stimuli and ensure steady-state EEG responses. The average luminance was 40.5 cd/m^2^, and the Michelson contrast was 99.1%.

**Fig. 1:**
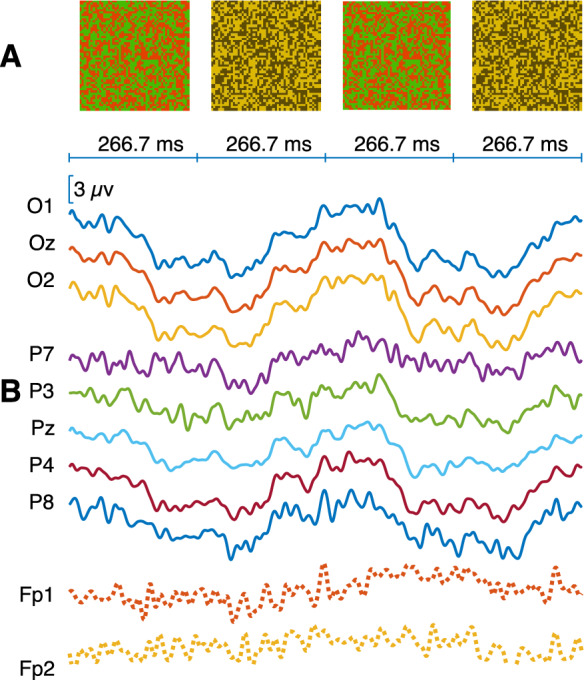
Representative example of averaged multi-channel VEP responses evoked by a dynamic random dot correlogram (DRDC). **A** Illustration of the different phases (each displayed for 266.7 ms) of the full-field DRDC stimulus alternating at 1.875 cps frequency. The first and third images illustrate the correlated phase featuring yellow and black dots that appear as identical patterns for both eyes when viewed through anaglyph glasses. The second and fourth images correspond to the anticorrelated phase, because they are composed of red and green dots resulting in the perception of opposite contrast patterns by the two eyes. **B** Averaged VEP responses for the 10 EEG channels recorded in participant S215. Solid lines represent significant responses based on T^2^_circ_ statistics, and dotted lines indicate nonsignificant responses

Second, two types of cyclopean stimuli were used: dynamic random dot correlograms (DRDC) [2] and dynamic random dot stereograms (DRDS) [13]. In DRDC stimuli, correlated and anticorrelated states alternated, where the correlated state was characterized by equal contrast of the dots in the two eyes (correlation coefficient + 1), whereas the anticorrelated state they had opposite contrast (correlation coefficient − 1). As these stimuli occupied the entire screen and apart from the random dots, no other spatial pattern could be discerned (Fig. 1 Panel A), we call it a full-field stimulus. To minimize interocular crosstalk, dot colors were calibrated according to prior research [25]. The average luminance was 2.17 cd/m^2^, and the Michelson contrast was 19.3% when viewed through anaglyphic glasses. We measured the luminance of stimuli by using an ILT1700 photometer (International Light Technologies, Peabody, MA) equipped with a SED033 detector, Y2 filter and R input optic.

**Fig. 2:**
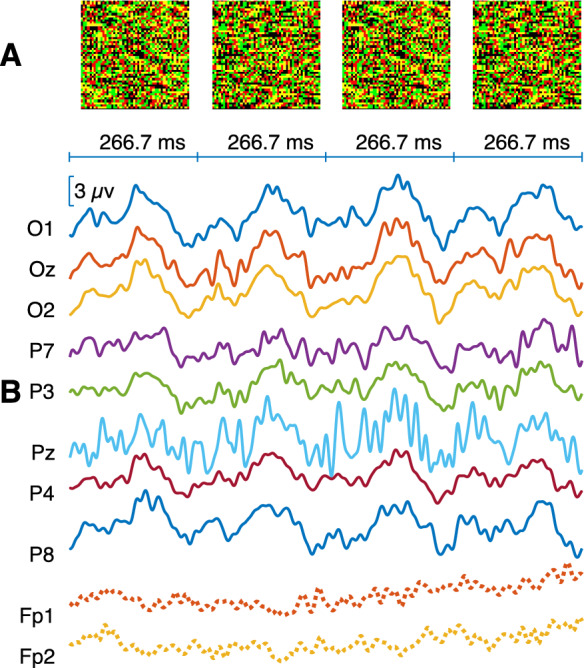
Representative example of averaged multi-channel VEP responses evoked by a dynamic random dot stereogram (DRDS) checkerboard stimulus. **A** Illustration of the different phases of the DRDS stimulus alternating at 1.875 cps frequency. Each frame contains red, green, yellow and black dots in equal proportions. In the above illustration, the bright and dark dots are arranged to create the illusion of a 6 × 6 checkerboard pattern, with squares appearing either in front of or behind the plane of fixation. The disparity of the squares alternated its sign from phase to phase every 266.7 ms, creating the illusion of depth reversal. **B** Averaged VEP responses for the 10 EEG channels recorded in participant S227. Solid lines represent significant responses based on T^2^_circ_ statistics, and dotted lines indicate nonsignificant responses

For DRDS stimuli, disparity-defined checkerboards were presented using a single pair of reversing disparities (± 7’) with a check size of 168’ (Fig. 2). The red dots were set to maximum achievable luminance, while the green dots were adjusted to match this level, resulting in an average luminance of 1.63 cd/m^2^ and a Michelson contrast of 91.7% through the anaglyphic glasses.

Dot size was 3.5’ both for DRDC and DRDC. Anaglyphic channel separation and red-green filter glasses belonging to the TNO stereo test were used. Both stimuli were presented at three temporal frequencies: 0.9375, 1.875, and 3.75 cps. For instance, in a 1.875 cps DRDC stimulus, the cycle duration was 532 ms, comprising 266 ms each of the correlated and anticorrelated states.

### Study design

Each participant underwent a single measurement session, during which VEPs were recorded in response to the following stimuli:

1. Monocular, luminance-determined checkerboards (60’ and 15’).
2. DRDC and DRDS stimuli at temporal frequencies 0.9375, 1.875, and 3.75 cps (Table 1) under binocular, anaglyphic viewing conditions. (Figs. 1 and 2 Panel A and videos in the Electronic Supplementary material).
3. Control measurements were performed for each of the six cyclopean stimuli under the following conditions: monocular anaglyphic viewing with the right eye (green filter), monocular anaglyphic viewing with the left eye (red filter), and binocular viewing without anaglyphic glasses. In the latter case, channel separation did not occur, indicating that the stimulus was not stereoscopic; instead, both eyes received the same stimulus simultaneously. These controls were compared by T^2^_circ_ test to actual measurements to verify the dependency of responses on intact stereo vision.

Each cyclopean VEP recording lasted a minimum of 90 s, while recordings with luminance-determined checkerboards lasted 30 s. For each participants, we ensured that the order of stimulus presentation was randomized within the 45-min session, and the total number of right-eye, left-eye, and no-glasses control measurements were balanced throughout the entire study. Rest periods were provided as needed or when attention declined, as indicated by the presence of alpha waves or increased blinking in the EEG, monitored in real time by trained research staff through continuous visual inspection of the EEG recordings. Alpha activity was identified primarily over posterior scalp regions, while increased blink frequency was noted via frontal EEG channels. The duration and frequency of rest periods were not pre-specified but were tailored to individual needs.

### Data acquisition and analysis

EEG recordings were performed using the BrainVision Recorder software (Brain Products GmbH, Germany) with an actiCHamp EEG system (Brain Products GmbH) equipped with 64 channels and actiCAP slim electrodes. Electrode placement followed the 10–20 system and included the ground electrode (Cz), reference electrode (FCz), and 10 measurement electrodes (O1, Oz, O2, P7, P3, Pz, P4, P8, Fp1, Fp2) relevant to this experiment. Data were recorded at a sampling frequency of 10 kHz and filtered with a low cutoff at 0.05 Hz and a high cutoff at 1250 Hz.

Statistical analysis was performed using BrainVision Analyzer 2 (Brain Products GmbH, Germany) in conjunction with a custom MATLAB script, which can be found in the Supplementary Material (tcirc2_matlab_script.txt). To analyze evoked responses, the data were resampled to 960 Hz. This ensured an exact integer number of samples per cycle at the given stimulus frequency (0.9375 cps and its integer multiples), which is crucial for the subsequent fast Fourier transformation (FFT) to prevent spectral leakage during analysis [26].

During preprocessing, we applied a frequency filter with a high-pass filter set at 0.05 Hz and a low-pass filter set at 45 Hz cutoff. We excluded recording artefacts caused by eye movements or other sources, as follows. An automatic artefact rejection protocol was performed, whereby events where amplitude changes within a 200-ms EEG segment exceeded 100 μV were rejected along with the preceding 400 ms and following 200 ms segments. This effectively removed most of the blink artefacts. Further disturbances caused by potential muscle movements or other sources were removed manually by two of the authors (RJ and MBE).

For VEPs evoked by non-stereo checkerboard reversal stimuli, peak latencies were extracted *on the Oz electrode* using the *automatic peak detection algorithm of* BrainVision Analyzer 2 *(main positive peak between 70 and 120 ms), and the results were verified through visual inspection.* The inclusion of non-stereo checkerboard data served two purposes: First, to electrophysiologically confirm that participants had normal vision and typical EEG/VEP responses. Second, to assess signal quality in real time using a stimulus known to evoke a robust and easily identifiable response, even in the absence of statistical analysis.

For the analysis of ssVEPs evoked by cyclopean stimuli, the EEG data were segmented into non-overlapping epochs of 533 ms for the high-contrast checkerboard stimuli and 2133 ms for the stereo stimuli. Datasets containing fewer than 15 valid epochs following artifact removal were excluded from further analysis both in the case of the stereo and the control measurements. This number of epochs was expected to ensure moderate to high statistical power of the subsequent T^2^_circ_ statistical test [14].

To determine the presence of stimulus-related responses at each channel, we began by performing FFT on each epoch and extracting the Fourier components at the fundamental of the stimulus and its second harmonic. Throughout this paper, the fundamental frequency of the stimulus in cycles per second will be referred to as the 1 st harmonic and denoted as f1, whereas twice the fundamental frequency will be called the 2nd harmonic and denoted as f2.

The Fourier components obtained for each channel and response frequency were represented as sets of complex numbers, which can also be interpreted as two-dimensional vectors. The T^2^_circ_ statistical test [14] was then performed with a significance threshold of *p* < 0.01. The T^2^_circ_ test evaluates whether the average of a set of two-dimensional vectors differs significantly from the null vector. The null hypothesis for the T^2^_circ_ test states that the population mean vector in the complex plane is zero, i.e., that there is no consistent phase-locked response across epochs. The two-sample version of the T^2^_circ_ test was employed to compare vectors derived from a cyclopean ssVEP with those obtained from a control measurement using the same stimulus (Fig. 5).

**Fig. 3:**
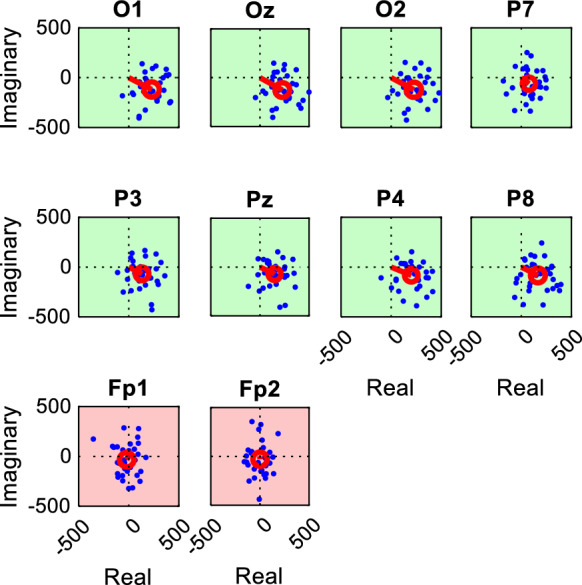
Representative T^2^_circ_ plots for a full-field DRDC stimulus (1.875 cps reversal rate, Participant S215) based on the same recording as that illustrated in Fig. 1 The blue dots represent the complex-valued Fourier coefficients for each 2133 ms epoch taken at the fundamental frequency of the stimulus, with their coordinates in the complex plane given by the real (*x*-coordinate) and imaginary (*y*-coordinate) parts (in relative units). The data points can also be interpreted as the tips of vectors whose lengths and angles represent the amplitude and phase of the response, respectively. A channel is considered to have a significant response if the 99% confidence interval (red circle) of the mean vector (red line) does not encompass the origin. Channels with significant responses are highlighted with a green background, while those without significant responses are shown with a red background

Figure 3 presents examples of T^2^_circ_ test results for a specific recording across various channels, illustrating the typical spatial distribution of Fourier component vectors associated with epochs significant or non-significant responses.

T^2^_circ_ values (as calculated by Eq. 1) are used to express signal-to-noise ratio of the complex-valued Fourier component vectors ${\mathrm{z}}_{\mathrm{j}}$ at the frequency of interest:

1 $${\mathrm{T}}_{{{\mathrm{circ}}}}^{2} = { }\left( {{\text{M }} - { }1} \right){ }\frac{{\left| {\overline{z}} \right|^{2} }}{{\mathop \sum \nolimits_{j} { }\left| {{\mathrm{z}}_{{\mathrm{j}}} - { }\overline{z}} \right|^{2} }}$$

where $\mathrm{M}$ is the number of independent estimates of the Fourier component, $\overline{z }$ is the empirical mean of the Fourier component estimates. The two-sample version of the T^2^_circ_ test was used to compare signals evoked by cyclopean and control stimuli [14].

Summary statistics for real valued data are reported as arithmetic means ± standard deviation unless stated otherwise.

The main outcome measure, *stimulus effectiveness*, was defined as the proportion of participants who exhibited a significant ssVEP response—at either the fundamental (f1) or second harmonic (f2) frequency—on any of the eight selected posterior channels. To further assess stimulus performance, we performed a receiver operating characteristic (ROC) analysis for each of the six conditions. Using the lowest T^2^_circ_ *p* value across the eight channels as a continuous predictor to discriminate between binocular (negative case) and monocular viewing (positive case) within subjects, we calculated the area under the curve (AUC), along with the optimal classification threshold, sensitivity, and specificity. To verify that our sample size was adequate for ROC analysis, we performed power analysis (MedCalc v23.2.6), assuming an expected AUC of 0.8, α = 0.05, β = 0.20 (80% power), a null hypothesis AUC of 0.5, and a 2:1 ratio of negative to positive cases. This analysis yielded a minimum requirement of 10 positive and 20 negative cases, which our dataset exceeded.

## Results

Each of the 22 examined participants fulfilled the following inclusion criteria: 1) successful completion of the TNO stereo test at the 120″ threshold indicating good to excellent stereo vision. 2) The average peak time of the binocularly recorded non-cyclopean checkerboard VEP was 99.7 ms (± 7.5 SD) and 104.6 ms (± 6.3 SD) for large and small check width, respectively. For monocular recordings (left and right), the average peak times on the Oz electrode were 102.3 ms (± 7.5 SD) and 104.7 ms (± 6.2 SD), respectively. The peak times of all participants were within the 90% confidence intervals reported by Thompson et colleagues [27] for the respective check sizes.

**Table 1:** Summary of statistical outputs and effectiveness values for the different cyclopean stimuli used in the study when considering all 8 electrodes

1	2	3	4	5	6	7	8	9	10	11
Stimulus	No. cases	Median No. epochs	Harmonic	Median of *p* values	IQR of *p* values	Median of T^2^_circ_ values	IQR of T^2^_circ_ values	No. significant cases	No. significant cases. for f1 or f2	Effectiveness
DRDC 0.9375 cps	21	30	f1	0.00063	0.00164	8.78	6.87	15	15	71%
			f2	0.00765	0.00316	5.36	0.50	3		
DRDC 1.875 cps	22	30	f1	0.00020	0.00132	9.94	8.98	21	22	100%
			f2	0.00099	0.00348	7.90	4.21	11		
DRDC 3.75 cps	22	29	f1	8.0714 × 10^–6^	0.00133	14.97	15.41	18	19	86%
			f2	0.00036	0.00197	10.27	9.28	14		
DRDS 0.9375 cps	21	34	f1	0.00422	0.00495	6.30	1.36	2	11	52%
			f2	0.00033	0.00185	9.16	4.74	10		
DRDS 1.875 cps	22	29	f1	0.00241	0.00216	6.86	1.013	2	18	82%
			f2	0.00004	0.00112	12.11	11.402	18		
DRDS 3.75 cps	22	33	f1	0.00208	0.00470	6.97	2.603	5	21	95%
			f2	0.00018	0.00199	10.39	14.69	20		

Values in columns 5–8 were pooled across all participants and all eight posterior electrode sites where significant responses were detected. Effectiveness (column 11) is defined as the proportion of the number of participants showing significant ssVEP on at least one of the channels O1, Oz, O2, P7, P3, Pz, P4 and P8 at the fundamental (f1, column 9) or first harmonic (f2, column 9) frequencies. Values in columns 3 and 5–8 are from recordings with significant responses

*DRDC* dynamic random dot correlograms, *DRDS* dynamic random dot stereograms, *f1* fundamental frequency, *f2* the second harmonic frequency, *cps* cycles per second, *No.* Number of, IQR interquartile range

### Derivation of the effectiveness measure for ssVEP responses evoked by cyclopean stimuli

The primary tool in assessing ssVEP responses was the T^2^_circ_ test. 

Table 1 summarizes the statistics for the six cyclopean stimuli under scrutiny. The p-values of the significant responses were well below the 0.01 threshold, confirming clear responses in most cases for all stimuli examined. The T^2^_circ_ value (Eq. 1) represents signal-to-noise ratio of the responses at the frequency in question; a higher T^2^_circ_ value indicates greater deviation from the null hypothesis, suggesting a stimulus-related response.

**Fig. 4:**
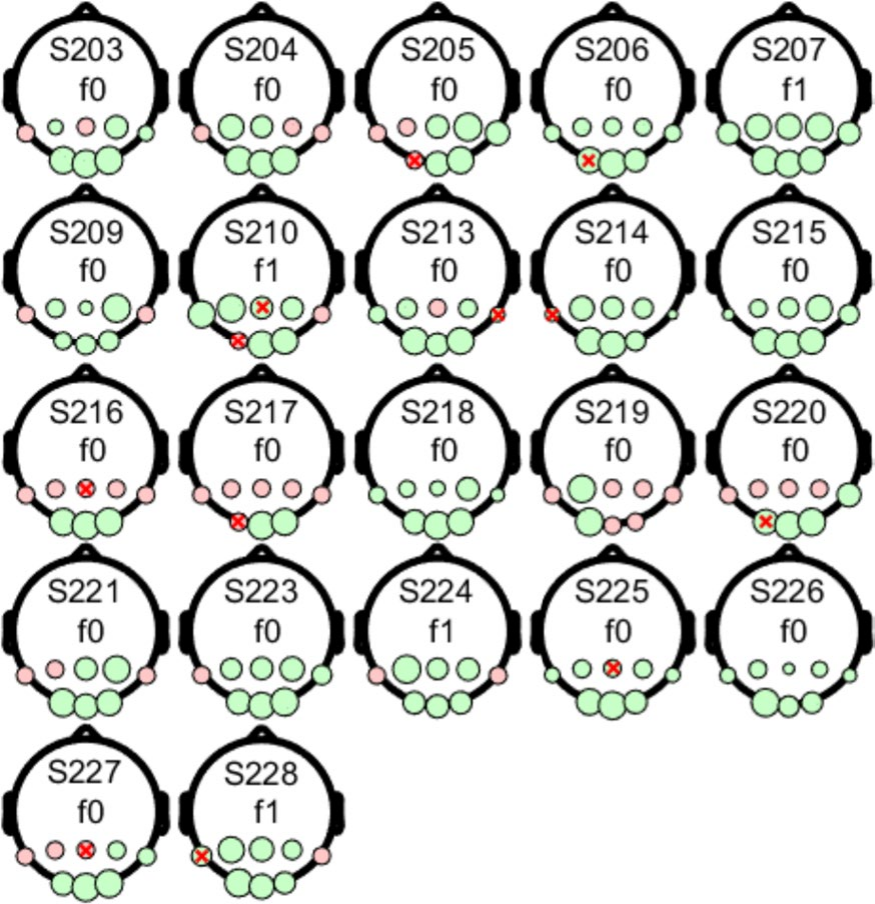
Summary of the participants’ responses evoked by the 1.875 cps DRDC stimulus. Each recording is represented by dots in a head model. The area of the green dots is proportional to the T^2^_circ_ value (see Materials and Methods) for the given recording at specific electrode positions, if the test result for that channel was significant. The dot representing the highest T^2^_circ_ value of the participant has the same normalized area on each head model. The labels (f1 or f2) indicate the harmonic at which more channels showed significance for the given recording. Red dots show electrode positions that were not significant in the given recording. While red x marks indicate electrodes that delivered noisy, uncertain signals as judged by visual inspection. Labels Sxxx are participant identifiers. The results for other test stimuli can be found in Figs. S1, S2, S3, S4 and S5 of the SI

The availability of multiple recording channels and the full frequency spectrum of each recording provide us with several options for assessing stereo vision. To consider the frequency component, 

Table 1 shows the number of significant recordings for both the fundamental frequency of the stimulus (f1) and the second harmonic frequency (f2). Consistent with the cyclic alternation of correlated and anticorrelated phases of the DRDC stimulus, f1 responses dominated the recordings, although a considerable number of recordings showed significant f2 responses. In certain participants, there were in fact, more channels showing significant f2 than f1 responses (see f1 or f2 labels in Fig. 4). Conversely, the two phases of the DRDS checkerboard stimulus were equal in terms of all spatially averaged stimulus parameters, and only local reversals of disparity could be perceived. Accordingly, the second harmonic frequency dominated the responses, although in certain cases, the f1 response was also significant (Table 1) or even dominant (see f1 or f2 labels in SI Figs. S3, S4, S5). Therefore, we decided to take both f1 and f2 frequency components into consideration, when assessing the effectiveness of stimuli.

To understand the variability of responses by channel location, one may find the representation of Fig. 4 helpful. The figure illustrates the significance of responses at f1 and f2 frequencies, as well as the relative T^2^_circ_ values for all participants, with the channels arranged on head models. For the 1.875 cps DRDC illustrated there, significant responses in individual participants were predominantly observed in the O1, Oz, and O2 channels. However, responses were also frequently significant on one or more of the P7, P3, Pz, P4, and P8 channels. Fp1 and Fp2 channels were not considered here, as they only served for the detection of gross eye movements.

Similar head model images for the remaining five stimuli are provided in SI Figs. S1, S2, S3, S4 and S5. They not only show similar variability in the dominant channel location but also illustrate cases where significant stereo responses appeared on the parietal channels but not on the occipital ones.

The upshot of these observations is that the dominant frequency and dominant location of stereo responses varies by individual as well as by the exact set of stimulus parameters. To address the resulting challenge of comparing the suitability of stereo stimuli for the assessment of stereo vision, we derived the metric of stimulus effectiveness based on the ability to elicit a significant response on any of channels O1, Oz, O2, P7, P3, Pz, P4 and P8 at the fundamental or first harmonic frequencies. Accordingly, we calculated the ratio of participants showing significant responses on any of those eight channels at f1 or f2 over the total number of participants (Table 1, column 11).

### Comparing the effectiveness and response patterns of cyclopean stimuli

Based on the metric derived above, the most effective stimulus was the 1.875 cps DRDC, which evoked a significant response in 100% of participants. The second most effective stimulus was the 3.75 cps DRDS, with a 95% effectiveness in our stereo normal participants. In contrast, the slower, 0.93 cps stimuli performed less well, with effectiveness values of 71% for DRDC and 52% for DRDS.

To model the results expected when using a standard clinical VEP setup, which typically uses only three to five electrodes, we also assessed stimulus effectiveness using only three occipital electrodes (see SI Table 2). The 1.875 cps DRDC stimulus remained highly effective, eliciting significant responses in all cases. However, the effectiveness of the 1.875 cps and 3.75 cps DRDS stimuli declined considerably in this setting, achieving approx. 70% effectiveness. Stimuli under 1 cps are generally less likely (62% and 24% for DRDC and DRDS, respectively) to evoke a VEP that can be detected by T^2^_circ_ test, so we do not recommend low frequencies in this setting.

Regarding the number [1–8] and the distribution of the significant responses over the different channels, a stimulus-specific pattern could be observed. For the 1.875 cps DRDC stimulus (Fig. 4), all 22 participants showed a significant response at Oz, O1 and O2, including 7 who showed significance in each of the 8 electrodes, although with typically lower T^2^_circ_ values at parietal electrodes. Every participant exhibited a significant response on at least one of the occipital electrodes. It can generally be stated that DRDS stimuli (Figs. 4, S3, S4) often elicited significant responses in the parietal electrode row, or only there, indicating a parietal involvement. For example, in the case of the most effective 3.75 cps DRDS (SI Fig. S5), out of the 21 individuals who gave significant responses, there were 6 who had significant responses in the parietal channels only. Finally, some of our participants exhibited slightly lateralized responses, sometimes to the point that the central locations Oz and Pz were non-significant (Figs. 4, S1, S2, S3, S4, S5).

As hinted above already, the frequency composition of the responses was also fundamentally influenced by the stimulus type. For DRDC, f1 responses were characteristic (Fig. 1), while additional f2 responses were only present in approx. 50% of the cases (Table 1). In the case of DRDSs, almost only f2 responses occurred (Fig. 2, Table 1). Significant f1-responses were rarely present, and there were only two participants who gave exclusively f1-resonse (Figs. S3, S5).

### Control measurements

For each measurement with cyclopean stimuli, control measurements were designed to judge the possibilities of false negative or false positive results. Based on the combinations of stimulus type (DRDC or DRDS) and the control condition (either monocular viewing or viewing without goggles), four kinds of controls were possible. Each control recording was evaluated by T^2^_circ_ tests for both the f1 and f2 frequencies. Therefore, the total number of cases reported below is always twice the number of channels analyzed.

**Fig. 5:**
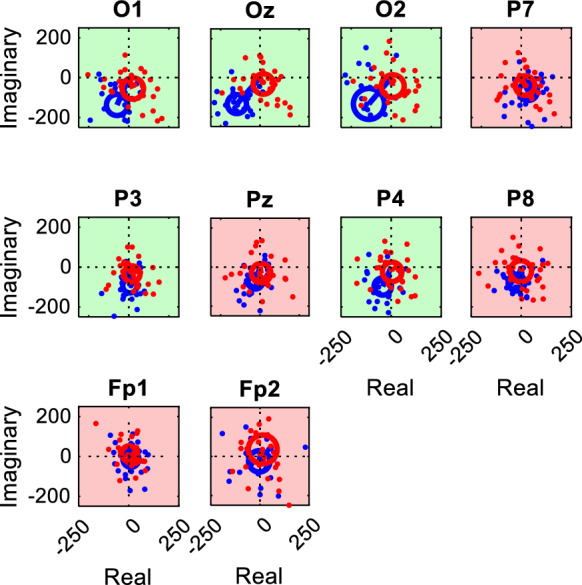
Representative T^2^_circ_ plots for the f1 frequency response to a full-field DRDC stimulus (3.75 cps reversal rate, Participant S206) and their monocular control (through the left eye). Fourier components of the epochs, mean vectors and confidence intervals are plotted in the same way as in Fig. 3, except that blue and red colors represent the binocular and control measurements, respectively. Every channel except Fp1 and Fp2 gave a significant binocular response (the blue confidence intervals do not encompass the origin). On channel O1, also the control measurement resulted in a spurious significant response (the origin is outside the red confidence interval), but the two-sample T^2^_circ_ test proved a significantly different response to the binocular stimulus. For each channel, green background signifies that the latter test proved significant difference from the control, whereas red background indicates that the two measurements did not differ significantly. Sometimes, small but significant binocular responses such as those on P7, Pz and P8, could not be differentiated from the control by the two-sample test

1. Viewing DRDC stimuli without glasses (Fig. 1) produces a perceptible exchange of color and potentially mean luminance, which might cause a significant ssVEP. This is important to be kept in mind, because a similar situation would occur if the observer’s visual field is not completely covered by the filter goggles, potentially resulting in a false positive judgement of their stereo vision. Indeed, among the 320 T^2^_circ_ tests, 173 (54%) showed a significant response in these control measurements.
2. When stimulus colors are well-calibrated, viewing DRDC stimuli monocularly (through either the red or green filter) should be perceived as random dynamic noise without any change between the correlated or anticorrelated phases, and thus, no significant VEP response is expected. A similar situation would occur if an amblyopic person viewed the stimuli, or if one recorded from a channel without stereo response, even if an individual has intact stereo vision. Among 720 T^2^_circ_ tests with this kind of control, only 5 yielded a significant (false positive) response (0.69%). However, in four of these five cases, ssVEP responses could be differentiated from the binocular responses by using the 2-sample T^2^_circ_ test [14]. In only one case (0.14%) the test was unable to distinguish the binocular response from the control. A representative false positive case is seen on channel O1 in Fig. 5.
3. Viewing DRDS stimuli without glasses results in no depth reversal, nor should a perceptible change in color or mean luminance occur (Fig. 2); therefore again, no significant ssVEP response was expected. Out of 368 T^2^_circ_ tests, only 14 showed a significant response in the control measurement. In 9 cases (2.45%), the two-sample test could not differentiate between the response to the stereo recording and the control.
4. Similarly, viewing DRDS stimuli monocularly (through either the red or green filter) results in no perceptible change in depth. Out of 704 T^2^_circ_ tests, 9 (1.28%) showed a significant response in the control measurement. In a single case (0.14%), the two-sample test failed to differentiate between the stereo and control responses.

We performed receiver operating characteristic (ROC) analysis to evaluate the sensitivity–specificity trade-offs associated with our ultimate goal of detecting stereopsis (Table 2). DRDC 1.875 cps and DRDS 3.75 cps yielded the highest AUCs (0.962 and 0.991, respectively), with optimal thresholds aligning closely with our predefined 0.01 cutoff for p-values. While DRDC stimuli generally achieved high sensitivity, they also showed increased false positive rates in the monocular control condition (up to 54%), especially at lower frequencies. In contrast, DRDS stimuli, particularly at 3.75 cps, demonstrated excellent specificity (95.5%) and more robust performance under control conditions. These findings highlight different trade-offs between sensitivity and specificity across stimulus types.

**Table 2:** Summary of ROC analysis of the different cyclopean stimuli used in the study

Stimulus	No. rec. binocular_ monocular	AUC	95% CI	Criterion *p* value (≤)	Sensitivity at criterion (%)	Specificity at criterion (%)	Effectiveness (Rado et al.)
DRDC 0.9375 cps	21_14	0.952	0.822–0.996	0.0225	100	85.7	71%
DRDC 1.875 cps	22_19	0.962	0.850–0.997	0.0056	94.7	100	100%
DRDC 3.75 cps	22_13	0.86	0.701–0.954	0.027	69.2	95.5	86%
DRDS 0.9375 cps	21_14	0.823	0.657–0.931	0.0183	92.9	61.9	52%
DRDS 1.875 cps	22_17	0.909	0.773–0.977	0.0019	100	72.7	82%
DRDS 3.75 cps	22_10	0.991	0.874–1.000	0.0100	100	95.5	95%

ROC curves were constructed using the lowest T2circ p-value across 8 EEG channels as a continuous predictor

The binary classification target was viewing condition (binocular vs. monocular), with the optimal threshold. *Effectiveness* denotes the main metrics used in the manuscript (% of participants (n = 22)) for whom at least one EEG channel showed a significant T^2^_circ_ response (*p* < 0.01) under binocular viewing.)

*AUC* area under the curve, *CI* confidence interval, *DRDC* dynamic random dot correlograms, *DRDS* dynamic random dot stereograms, *cps* cycles per second, *No.* number of, *rec.* recording

## Discussion

### Previous electrophysiological studies utilizing random dot correlograms and stereograms in humans

Random dot correlograms and stereograms (DRDC and DRDS) isolate the stereoscopic visual mechanism: they only evoke a response if stereopsis is functional. Their effectiveness does, however, depend on many parameters.

There are only a few studies that systematically examine the applicability of DRDC and DRDS stimuli in humans. In Table 3, we summarize key test parameters from notable publications. Béla Julesz, who first described the application of DRDCs in human VEP studies [2], reported that DRDCs offer several advantages over DRDSs, including simpler stimulus generation and a greater absolute magnitude of evoked responses. Additionally, DRDC functions effectively at higher stimulus frequencies (12 rps). They also highlighted its insensitivity to head tilt, the necessity of a large stimulus size, and the differences in response periodicity between DRDCs and DRDSs.

**Table 3:** Stimulus parameters utilized in previous DRDC and/or DRDS VEP studies reported in the literature

Author, Year	Julesz et al. [2]	Petrig et al. [6]	Bergua et al. [18]	Skrandies [17]	Markó et al. [19]
Participant healthy/impaired stereo vision	4/0	17/0	22/22	20/0	16/0
Stimulus (DRDC, DRDS)	RDC/RDS	DRDC/DRDS	DRDS	DRDS	DRDC
Channel separation	Anaglyphic	Anaglyphic	Shutter	Anaglyphic	Anaglyphic
Goggles	Kodak Wratten red and green filters	Kodak Wratten colour filters (Nos. 55 and 26)	Electric liquid crystal shutter glasses	Red/green goggles	R26 low-pass and YG09 band-pass gelatine
Stimulus pattern	DRDC full-field correlation reversal, DRDS checkerboard depth onset-offset	DRDC full-field correlation reversal, DRDS checkerboard depth onset-offset	Checkerboard depth onset-offset	Checkerboard depth alternation	Full-field correlation reversal
Field size (°)	38.6 × 30.2	82 × 68	28.4 × 21.9	17.1 × 13.4	40 × 30
Viewing distance [mm]	2500	1000	750	1500	1000
Check size [arcmin]	330 × 330	280 × 280	132 × 164	90	Not applicable
Dot size [arcmin]	13.8 × 6.9	20 × 18	1.38 × 1.84	3.5′	7.5
Dot density [%]	100	100	0.4	No data	100
Stimulus rate [cps]	1 and 3	2.5 cps	1	4	1.875
Duration of phases [ms]	500/500 and 166.7/166.7	200/200	500/500	125/125	266.7/266.7
Monitor type	Advent VideoBeam 1000 A projection system	Advent VideoBeam 1000 A projection system	two Eizo F77 CRT monitors	Hitachi CRT monitor	Samsung SyncMaster 957 MB CRT monitor
Monitor refresh rate (Hz)	60 interlaced	50	120	48	60
Mean luminance [cd/m^2^]	No data	8.25	5.5	20	2,89 ± 0,18
Contrast [%]	Maximum black and white	82%	No data	No data	5.5–80 in 10 steps
Disparity [arcmin]	27.6’ far	40’ far	9, 13, 22, 35, 40	7, 10.5, 14, 17.5, 21, 24.5’	Not applicable
No. active electrodes	1	1	1	7	1
Active electrode position	2 cm above the inion	2 cm above the inion	5 cm above inion	Oz, O1, O2, P7, P8, PO7, PO8	Oz
Reference electrode position	Halfway between vertex and nasion	30 percent nasion-inion distance	Right ear	Fz	Fz
Gnd electrode position	Forehead	Vertex	Left ear	Pz	Cz
Epoch duration [ms]	1000	800	1000	1000	2133
Bandpass filter [Hz]	0.1 and 100	0.5 and 40	0.5 and 70 Hz	0.1 and 70 Hz	0.5 and 250 Hz
Presentation duration (s)	12 sessions * 75 s	40	No data	300	70
Minimum No. of epochs	75	50	50	No data	10

*Arcmin* minutes of arc, *cd/m*^*2*^ candela per square meter, *CRT* cathode ray tube; *DRDC* dynamic random dot correlograms, *DRDS* dynamic random dot stereograms, *cps* cycles per secundum, *Hz* Hertz, *ms* millisecond, *No.* number of, *VEP* visual evoked potentials

In their study on infants, Petrig et al. [6] applied DRDC full-field correlation reversal and DRDS checkerboard depth onset-offset stimuli, reporting that both evoked responses with comparable amplitudes in early development. Braddick [28] found that the mean age for the first detectable response was correlated between the two tests, with no significant difference (correlogram: 70 days; stereogram: 75 days). This aligns closely with the age at which the first signs of stereopsis are observed in psychophysical tests [8].

Skrandies' seven-channel study demonstrated responses to DRDS checkerboard reversal at five temporo-occipital channels, specifically at the first harmonic frequency. Psychophysically estimated stereo thresholds were lower than electrophysiologically determined thresholds. Furthermore, they showed that strabismic—but not stereo blind—patients exhibited higher-than-normal thresholds as determined by DRDS-VEP [11]. Additionally, they found the largest response amplitude approximately 4.25 cm (± 1.6 cm) from the inion [3].

Bergua et al. observed no significant differences in VEP peak times in response to DRDS onset-offset stimuli with disparities between 9.0′ and 40′ in healthy individuals. However, in patients with glaucoma, peak times were significantly delayed [18].

In two previous studies, we examined the effects of luminance and contrast changes on DRDC responses. We found that response amplitudes remained stable across a broad range of contrast (5.5–80%) and luminance (0.06–5.5 cd/m^2^). However, significant reductions in response amplitude were observed with decreases in luminance, but none with decreasing contrast [19, 20]. These findings support the magnocellular origin of DRDC responses.

Regarding the specific stimulus parameters, the studies listed in Table 3 are in broad agreement, but they typically derive from exploration that is not systematically described in the papers. Together with our pilot studies (see SI Table 1), we explored a large range of various parameters with the specific aim of optimizing stereo VEPs. Our current data suggests that the most likely stimulus to evoke an ssVEP response is the full-field (spatially homogeneous) DRDC where dot correlation is modulated at around 1.875 cps (Table 1). Our DRDS stimulus at 3.75 cps produced only slightly fewer significant responses, but correlograms were in general, superior to stereograms. DRDC stimuli were also superior in that a stable response was obtained when using only the three occipital electrodes (SI Table 2). As discussed below, the location of EEG channels also turned out to be highly relevant to optimizing the electrophysiological detection of stereoscopic function.

### The source of correlogram and stereogram responses

Our findings are intriguing because the spatio-temporal patterns encoded in DRDCs are perceptually less salient than disparity defined patterns of the DRDSs [2]. Despite the higher subjective salience of stereograms, they produced less reliable ssVEP responses than correlograms. These differences likely reflect distinct neural pathways. It has been suggested that the processing of stereo disparity [29, 30] as well as the processing of DRDCs [19] rely on the activity of the magnocellular visual pathway, which dominates the activity of the dorsal stream of extrastriate visual cortex. Work by Cummings et al. [5] has pointed out differences between cortical regions in the processing of disparity by employing anti-correlated stereograms. In low-level visual areas V1 and V2, disparity tuned neurons give responses to both correlated and anti-correlated stereograms, although their disparity tuning is opposite. Binocular neurons of the dorsal stream [31] also respond to anticorrelated stereograms. However, this is in contrast with conscious experience, because disparity in anti-correlated stereograms cannot be perceived. The perception of observers is better reflected in the activity of extrastriate areas of the ventral stream, which are less responsive or not responsive at all to anti-correlated stereograms. Thus, the stronger DRDC than DRDS responses in the VEP may be explained by the presence of large neuronal populations modulated by changes in binocular correlation in occipital and dorsal visual cortical regions.

Our data also point to differences in spatial localization. Even though stereo stimuli primarily evoked responses at O1, Oz, and O2 electrode locations, we regularly found significant stereo responses on parietal channels P7, P3, P4, P8, and Pz. In the case of DRDS, these channels were more prominent, or in some participants, the only source of significant stereo response (SI Figs. S3, S4, S5). To objectively assess stereopsis by VEP, it seems therefore prudent to record activity from parietal as well as occipital channels.

In our adult participants, the DRDC stimulus typically elicited a fundamental frequency (f1) response, whereas the DRDS stimulus evoked a predominantly second harmonic (f2) response. Often, significant responses on both harmonics occurred. This difference is well explained by the nature of the stimuli: a full-field DRDC involves the alternation of correlated and anticorrelated dot patterns, where the two phases drive opposite responses [2, 20]. In contrast, the two phases of a checkerboard reversal stimulus (whether luminance or disparity defined), are in terms of spatial average, equal. The pronounced f2 response is driven by the local changes, which occur twice during the cycle, and the two reversals are equal in terms of the net direction of change, which is 50% near-to-far and 50% far-to-near every time.

In summary, EEG responses to cyclopean stimuli vary depending on stimulus attributes, spatial location, and response frequency, and these effects may also differ between individuals. Our current focus was to describe this complexity and to provide a method for identifying cyclopean responses in the face of such variability. Nevertheless, further analysis using more complex statistical models that account for the relationships and interactions among these variables may help uncover the underlying principles governing the spatial and spectral distribution of cyclopean VEPs in the future.

### Considerations for standardizing stereopsis assessment by ssVEP

VEPs have been used routinely in the diagnosis of visual pathway disturbances in humans [24]. Recently, a standard protocol employing periodic stimuli and steady-state potentials has been devised to estimate visual acuity [16]. Our findings provide a foundation for optimizing stereopsis assessments in both research and clinical applications. A key challenge to address is the individual variability in localization and frequency composition of the evoked response, which exist even in a population with intact stereopsis. We suggest that standard VEP procedures need to be amended in two ways for this purpose. First, it is necessary to consider activity from occipital as well as parietal channels. Second, both fundamental and second harmonic responses must be considered.

We used CRT monitors for stimulus display primarily because they do not exhibit luminance transients during changes in pixel intensity, and they maintain stable color and luminance across a wide range of viewing angles. These characteristics comply with current ISCEV requirements for stimulus devices [24] and were instrumental in ensuring that, after proper calibration, our DRDC and DRDS stimuli were free of monocular cues [25]. We chose the anaglyph technique for achieving dichoptic separation because of its simplicity and low cost, acknowledging that perfect channel separation is more challenging to achieve than with Wheatstone stereoscopy. In our setup, this limitation was addressed by rigorous calibration of the monitor’s colors based on empirical photometric characterization of our CRT + filters system, applying the same methodological principles described by Radó et al. [25]. While Radó et al. used an LED display with red–green glasses, their calibration approach is not display-specific and can be readily adapted to any filter-based three-dimensional display once the spectral and luminance characteristics of the actual setup are measured. Following calibration, both DRDC and DRDS stimuli were tested for residual monocular cues. Our control measurements confirmed that DRDC stimuli, although more effective at eliciting responses than DRDS, are also more sensitive to imperfect calibration, which may produce false positive VEP responses [25]. These occasional monocular false positives differed statistically (in amplitude and phase) from genuine binocular responses (Fig. 5). Anaglyphic DRDCs can also evoke significant binocular ssVEP responses when the goggles are removed (Figs. 1, 5, also see [2]), or when parts of the stimulus remain visible around the goggles. These findings highlight the need for stringent calibration and physical stimulus occlusion when using DRDCs. By contrast, the DRDS stimuli we used were less susceptible to spurious monocular responses caused by imperfect color calibration or removal of the filter goggles. However, detecting a stereo response to DRDSs likely requires recordings from at least six electrodes, as these stimuli predominantly activate parietal cortical areas.

Control measurements without goggles, as well as monocular control conditions, are essential for verifying the reliability of the stimulation and recording setup at the outset. Such controls should also be repeated regularly to ensure that calibration remains uncompromised over time, for example, due to aging of the stimulus screen or degradation of the filter glasses.

Our ROC analysis underscores the importance of balancing sensitivity and specificity when selecting stereoscopic stimuli for clinical or research use. DRDC stimuli -though highly sensitive- were more susceptible to false positives under monocular viewing, likely due to luminance artefacts or incomplete channel separation. DRDS stimuli, especially at 3.75 cps, offered greater specificity and robustness, making them more suitable for clinical environments where calibration is limited. Based on these findings, a two-stage testing approach could be considered: initial screening with DRDS, followed by DRDC confirmation under more controlled conditions.

Attentional fatigue is a potential factor that can both deteriorate and add further complexity to ssVEP recordings [32]. Although rest periods were provided based on signs of attentional decline in the current study, their duration and frequency were not formally monitored or correlated with signal quality. Therefore, residual differences in attention or engagement may still have contributed to inter-individual variability in ssVEP responses. Recent theoretical studies [33, 34] have proposed an analytical framework with the potential to identify systematic departures from the circular symmetry of ssVEP Fourier components [35], which may arise due to fluctuating attention during recording. This approach could be incorporated into future protocols for monitoring participants' attention, complementing conventional EEG-based fatigue markers such as increased alpha-band power.

The development of stereopsis is an experience dependent process [7, 36, 37] extending over years in early childhood [6, 8, 28]. Age-related changes in ssVEP responses have been found in our previous studies [7, 21] showing that DRDC stimuli in young children elicit dominantly f2 harmonic responses with an age-dependent phase change, reflecting latency decreases. After the age of 6 months, an increasing f1 component appears in the responses. Further research is required to optimize stimuli, recording configuration and analysis methods beyond the relatively homogeneous, healthy participant population of the present study.

The main limitation of the study is that, due to the use of multiple electrode sites and the analysis of different harmonic components across six distinct stimuli, it was not possible to define a single output variable for a predefined statistical test. Consequently, a custom metric (*effectiveness*) was developed to compare stimulus performance, and the specific statistical power could not be calculated. However, we believe that in future translational studies, ROC analysis may serve as an appropriate statistical approach to distinguish individuals with impaired versus intact binocular vision. The current sample size, which exceeded the minimum required to detect an AUC of 0.8 with 80% statistical power, may provide a valid reference for future investigations involving clinical populations with reduced stereopsis, where comparisons against a control group will remain essential.

## Supplementary Information

Below is the link to the electronic supplementary material.

Supplementary file1 (DOCX 425 KB)

Supplementary file2 (AVI 4260 KB)

Supplementary file3 (AVI 14774 KB)

Supplementary file1 (TXT 7 KB)

## Abbreviations

’: Arcmin minutes of arc
”: Arcsec seconds of arc
cd/m^2^: Candelas per square meter
cps: Cycles per second
CRT: Cathode ray tube
DRDC: Dynamic random dot correlograms
DRDS: Dynamic random dot stereograms
f1: Fundamental frequency
f2: Second harmonic frequency, i.e. 2 × f1
EEG: Electroencephalography
FFT: Fast Fourier transform
IQR: Interquartile range
rps: Reversal per secundum
SD: Standard deviation
ssVEP: Steady-state visual evoked potentials
T^2^_circ_: Circular T-squared
VEP: Visual evoked potentials

## Declarations

### Statement on the welfare of animals

This article does not contain any studies with animals conducted by the authors.

## References

[1] Cumming BG, Parker AJ (1994) Binocular mechanisms for detecting motion-in-depth. Vis Res 34(4):483–495. 10.1016/0042-6989(94)90162-7 8303832 10.1016/0042-6989(94)90162-7

[2] Julesz B, Kropfl W, Petrig B (1980) Large evoked potentials to dynamic random-dot correlograms and stereograms permit quick determination of stereopsis. Proc Natl Acad Sci USA 77(4 I):2348–2351. 10.1073/pnas.77.4.2348 6769126 10.1073/pnas.77.4.2348 PMC348712

[3] Skrandies W, Vomberg HE (1985) Stereoscopic stimuli activate different cortical neurones in man: electrophysiological evidence. Int J Psychophysiol 2(4):293–296. 10.1016/0167-8760(85)90007-8 3997617 10.1016/0167-8760(85)90007-8

[4] Janssen P, Vogels R, Orban GA (1998) Assessment of stereopsis in rhesus monkeys using visual evoked potentials. Doc Ophthalmol 95(3–4):247–255. 10.1023/A:1001848105993 10532408 10.1023/a:1001848105993

[5] Cumming BG, Parker AJ (1997) Responses of primary visual cortical neurons to binocular disparity without depth perception. Nature 389(6648):280–283. 10.1038/38487 9305841 10.1038/38487

[6] Petrig B, Julesz B, Kropfl W, Baumgartner G, Anliker M (1981) Development of stereopsis and cortical binocularity in human infants: electrophysiological evidence. Science 213(4514):1402–1405. 10.1126/SCIENCE.7268443 7268443 10.1126/science.7268443

[7] Jandó G, Mikó-Baráth E, Markó K, Hollod́y K, Tör̈ok B, Kovacs I (2012) Early-onset binocularity in preterm infants reveals experience-dependent visual development in humans. Proc Natl Acad Sci USA 109(27):11049–11052. 10.1073/pnas.1203096109 22711824 10.1073/pnas.1203096109 PMC3390849

[8] Birch E, Petrig B (1996) FPL and VEP measures of fusion, stereopsis and stereoacuity in normal infants. Vis Res 36(9):1321–1327. 10.1016/0042-6989(95)00183-2 8711910 10.1016/0042-6989(95)00183-2

[9] Eizenman M, Westall CA, Geer I et al (1999) Electrophysiological evidence of cortical fusion in children with early-onset esotropia. Investig Ophthalmol Vis Sci 40(2):354–362 9950593

[10] Westall CA, Eizenman M, Kraft SP, Panton CM, Chatterjee S, Sigesmund D (1998) Cortical binocularity and monocular optokinetic asymmetry in early- onset esotropia. Invest Ophthalmol Vis Sci 39(8):1352–1360 9660483

[11] Skrandies W (2001) The processing of stereoscopic information in human visual cortex: psychophysical and electrophysiological evidence. Clin EEG Neurosci 32(3):152–159. 10.1177/155005940103200310 10.1177/155005940103200310 11512379

[12] France LW (2006) Evidence-based guidelines for amblyogenic risk factors. Am Orthopt J 56(1):7–14. 10.3368/aoj.56.1.7

[13] Julesz B (1960) Binocular depth perception of computer-generated patterns. Bell Syst Tech J 39(5):1125–1162. 10.1002/j.1538-7305.1960.tb03954.x

[14] Victor JD, Mast J (1991) A new statistic for steady-state evoked potentials. Electroencephalogr Clin Neurophysiol 78(5):378–388. 10.1016/0013-4694(91)90099-P 1711456 10.1016/0013-4694(91)90099-p

[15] Meigen T, Bach M (1999) On the statistical significance of electrophysiological steady-state responses. Doc Ophthalmol 98(3):207–232. 10.1023/A:1002097208337 10945442 10.1023/a:1002097208337

[16] Hamilton R, Bach M, Heinrich SP et al (2021) ISCEV extended protocol for VEP methods of estimation of visual acuity. Doc Ophthalmol 142(1):17–24. 10.1007/S10633-020-09780-1/FIGURES/1 32676804 10.1007/s10633-020-09780-1 PMC7906925

[17] Skrandies W (2009) Assessment of depth perception using psychophysical thresholds and stereoscopically evoked brain activity. Doc Ophthalmol 119(3):209–216. 10.1007/s10633-009-9202-9 19856007 10.1007/s10633-009-9202-9

[18] Bergua A, Horn FK, Martus P, Jünemann AM, Korth M (2004) Stereoscopic visual evoked potentials in normal subjects and patients with open-angle glaucomas. Graefes Arch Clin Exp Ophthalmol 242(3):197–203. 10.1007/s00417-003-0797-3 14663591 10.1007/s00417-003-0797-3

[19] Markó K, Kiss HJM, Mikó-Baráth E et al (2009) Contrast independence of dynamic random dot correlogram evoked VEP amplitude. J Vis 9(4):1–10. 10.1167/9.4.8 19757917 10.1167/9.4.8

[20] Markó K, Mikó-Baráth E, Kiss HJ, Török B, Jandó G (2012) Effects of luminance on dynamic random-dot correlogram evoked visual potentials. Perception 41(6):648–660. 10.1068/p7042 23094455 10.1068/p7042

[21] Mikó-Baráth E, Markó K, Budai A, Török B, Kovacs I, Jandó G (2014) Maturation of cyclopean visual evoked potential phase in preterm and full-term infants. Invest Ophthalmol Vis Sci 55(4):2574–2583. 10.1167/iovs.14-13906 24644050 10.1167/iovs.14-13906

[22] Brainard DH (1997) The psychophysics toolbox. Spat Vis 10(4):433–436. 10.1163/156856897X00357 9176952

[23] Pelli DG (1997) The videotoolbox software for visual psychophysics: transforming numbers into movies. Spat Vis 10(4):437–442. 10.1163/156856897X00366 9176953

[24] Odom JV, Bach M, Brigell M et al (2016) ISCEV standard for clinical visual evoked potentials: (2016 update). Doc Ophthalmol 133(1):1–9. 10.1007/s10633-016-9553-y 27443562 10.1007/s10633-016-9553-y

[25] Radó J, Sári Z, Buzás P, Jandó G (2020) Calibration of random dot stereograms and correlograms free of monocular cues. J Vis 20(4):3 32271895 10.1167/jov.20.4.3 PMC7405724

[26] Bach M, Meigen T (1999) Do’s and don’ts in Fourier analysis of steady-state potentials. Doc Ophthalmol 99(1):69–82. 10.1023/A:1002648202420 10947010 10.1023/a:1002648202420

[27] Thompson DA, Mikó-Baráth E, Hardy SE, Jandó G, Shaw M, Hamilton R (2023) ISCEV standard pattern reversal VEP development: paediatric reference limits from 649 healthy subjects. Doc Ophthalmol 147(3):147–164. 10.1007/s10633-023-09952-9 37938426 10.1007/s10633-023-09952-9 PMC10638119

[28] Braddick O (1996) Binocularity in infancy. Eye 10(2):182–188. 10.1038/eye.1996.45 8776447 10.1038/eye.1996.45

[29] Krug K, Cumming BG, Parker AJ (2004) Comparing perceptual signals of single V5/MT neurons in two binocular depth tasks. J Neurophysiol. 10.1152/jn.00851.2003 15102899 10.1152/jn.00851.2003

[30] Roy JP, Komatsu H, Wurtzi RH, Wurtz RH (1992) Disparity sensitivity of neurons in monkey extrastriate area MST. J Neurosci 12:2478–2492 1613542 10.1523/JNEUROSCI.12-07-02478.1992 PMC6575856

[31] Mishkin M, Ungerleider LG, Macko KA (1983) Object vision and spatial vision: two cortical pathways. Trends Neurosci 6:414–417. 10.1016/0166-2236(83)90190-X

[32] Kashiwase Y, Matsumiya K, Kuriki I, Shioiri S (2012) Time courses of attentional modulation in neural amplification and synchronization measured with steady-state visual-evoked potentials. J Cogn Neurosci 24(8):1779–1793. 10.1162/JOCN_A_00212 22360591 10.1162/jocn_a_00212

[33] Norouzpour A, Klein SA (2022) A novel analytical method to measure intra-individual variability of steady-state evoked potentials; new insights into attention deficit. Biomed Signal Process Control 71:103109. 10.1016/J.BSPC.2021.103109

[34] Norouzpour A, Roberts TL, Klein SA (2023) A complementary note for the analytical method to estimate individual attention fluctuation using steady-state evoked potentials. Biomed Signal Process Control 81:104406. 10.1016/J.BSPC.2022.104406

[35] Mast J, Victor JD (1991) Fluctuations of steady-state VEPs: interaction of driven evoked potentials and the EEG. Electroencephalogr Clin Neurophysiol 78(5):389–401. 10.1016/0013-4694(91)90100-I 1711457 10.1016/0013-4694(91)90100-i

[36] Fawcett SL, Wang YZ, Birch EE (2005) The critical period for susceptibility of human stereopsis. Invest Ophthalmol Vis Sci 46(2):521–525. 10.1167/IOVS.04-0175 15671277 10.1167/iovs.04-0175

[37] Hubel DH, Livingstone MS (1987) Segregation of form, color, and stereopsis in primate area 18. J Neurosci 7(11):3378–3415. 10.1523/JNEUROSCI.07-11-03378.1987 2824714 10.1523/JNEUROSCI.07-11-03378.1987 PMC6569042

