# Supplementary material for: Optimizing cyclopean stimuli for the evaluation of stereo vision by steady-state visual evoked potentials
Source: Doc Ophthalmol. 2025 Oct 17;152(1):47–63. doi: 10.1007/s10633-025-10059-6 (PMC12948872; doi:10.1007/s10633-025-10059-6)
Supplement: Supplementary file 1 — Supplementary file1 (DOCX 425 KB) [file 10633_2025_10059_MOESM1_ESM.docx]

**Supplementary Material**

Dynamic random dot stimuli for the examination of stereo vision in VEP setup, Janos Radó, Eszter Mikó –Baráth, Péter Hegyi, Vanda Nemes, Jandó Gábor and Péter Buzas. Corresponding author Eszter Mikó –Baráth, Institute of Physiology, Medical School, University of Pécs, Pécs, Hungary; eszter.miko-barath@aok.pte.hu

**Supplementary Tables**

SI Table 1 The cyclopean stimuli used in the pilot study, their parameters, and the summary of statistical outputs. Three-channel effectiveness (column 10) is defined as the proportion of participants showing significant (by T^2^_circ_ test) ssVEP on a subset of the channels (O1, Oz, and O2) at either the fundamental or second harmonic frequencies. This measure of effectiveness was used as the basis for stimulus selection for the current study. Only stimuli with a three-channel effectiveness greater than 28% are included in this table. Eight-channel effectiveness (column 11), also applied in the current study, is defined as the proportion of participants showing significant ssVEP on at least one of the channels (O1, Oz, O2, P7, P3, Pz, P4, and P8) at the fundamental or first harmonic frequencies. Some values in this column appear twice because three-channel effectiveness was defined separately for both the fundamental (f1) and the first harmonic (f2) frequencies for the same stimulus. The dot size was 3.5’ for every stimulus, and the refresh rate was 60 Hz. Not every measurement was performed in each subject, therefore n is specified in each case.

| 1 | 2 | 3 | 4 | 5 | 6 | 7 | 8 | 9 | 10 | 11 |
| --- | --- | --- | --- | --- | --- | --- | --- | --- | --- | --- |
| Stimulus type | Pattern | Freq.  cps | Dot size [arcmin] | Disparity [arcmin] | Pattern size [arcmin] | Stimuli presentation | f1 or f2 harmonic | No.  Rec. | Effectiveness | |
|  |  |  |  |  |  |  |  |  | 3 channels | 8 channels |
| DRDC | Full- field | 0.94 | 5.46 |  |  | Correlation reversal | f1 | 11 | 82% | 91% |
| DRDC | Full- field | 0.94 | 2.73 |  |  | Correlation reversal | f1 | 5 | 80% | 100% |
| DRDC | Checkboard | 0.94 | 5.46 |  | 131 | Anticorrelation onset-offset | f1 | 12 | 78% | 91% |
| DRDC | Full- field | 0.94 | 5.46 |  |  | Correlation reversal | f1 | 34 | 74% | 85% |
| DRDC | Full- field | 0.94 | 4.09 |  |  | Correlation reversal | f1 | 12 | 69% | 91% |
| DRDS | Checkboard | 0.94 | 5.46 | ±5.46 | 131 | Depth onset-offset | f1 | 7 | 67% | 100% |
| DRDS | Checkboard | 2.66 | 2.73 | ±5.46 | 65 | Depth reversal | f2 | 6 | 67% | 83% |
| DRDC | Full- field | 0.94 | 6.82 |  |  | Correlation reversal | f1 | 12 | 61% | 90% |
| DRDC | Full- field | 3.75 | 5.46 |  |  | Correlation reversal | f2 | 10 | 60% | 100% |
| DRDC | Checkboard | 3.75 | 5.46 |  | 131 | Anticorrelation onset-offset | f2 | 6 | 56% | 100% |
| DRDC | Full- field | 1.88 | 5.46 |  |  | Correlation reversal | f1 | 9 | 52% | 100% |
| DRDC | Checkboard | 3.75 | 5.46 |  | 131 | Anticorrelation onset-offset | f1 | 6 | 50% | 100% |
| DRDS | Checkboard | 1.33 | 2.73 | ±5.46 | 65 | Depth reversal | f2 | 5 | 47% | 80% |
| DRDS | Checkboard | 0.94 | 5.46 | ±5.46 | 131 | Depth reversal | f2 | 10 | 40% | 50% |
| DRDC | Checkboard | 1.88 | 5.46 |  | 65 | Anticorrelation onset-offset | f1 | 7 | 33% | 50% |
| DRDS | Checkboard | 0.94 | 5.46 | ±5.46 | 65 | Depth reversal | f2 | 12 | 33% | 55% |
| DRDC | Full- field | 0.94 | 5.46 |  |  | Correlation reversal | f2 | 11 | 30% | 91% |
| DRDC | Full- field | 0.94 | 4.09 |  |  | Correlation reversal | f2 | 12 | 28% | 91% |
| DRDC | Full- field | 1.33 | 2.73 |  |  | Correlation reversal | f2 | 6 | 28% | 83% |

Arcmin minutes of arc, DRDC dynamic random dot correlograms, DRDS dynamic random dot stereograms, cps cycles per secundum, No. Number of

SI Table 2 Summary of statistical outputs and effectiveness values for the different cyclopean stimuli used in the current study as presented in

Table 1, except that only the 3 occipital electrodes are considered. Thus, the effectiveness (column 11) is defined as the proportion of the number of participants showing significant ssVEP on at least one of the channels O1, Oz and O2 at the fundamental (f1, column 8) or first harmonic (f2, column 9) frequencies. Values in columns 2-6 are from recordings with significant responses.

| 1 | 2 | 3 | 4 | 5 | 6 | 7 | 8 | 9 | 10 | 11 |
| --- | --- | --- | --- | --- | --- | --- | --- | --- | --- | --- |
| Stimulus | Median of *p* values | IQR of *p* values | Median of T^2^_circ_ values | IQR of T^2^_circ_ values | Median No. epochs | No. recordings | No. significant rec. for f1 | No. significant rec. for f2 | No. significant rec. for f1 or f2 | Effectiveness (3 channels) |
| DRDC 0.9375 cps | 0.00061 | 0.00284 | 8.7 | 6.9 | 29 | 21 | 13 | 3 | 13 | 62% |
| DRDC 1.875 cps | 0.00014 | 0.00166 | 10.3 | 8.2 | 31 | 22 | 21 | 9 | 22 | 100% |
| DRDC 3.75 cps | 0.00002 | 0.00044 | 14.0 | 9.5 | 29 | 22 | 18 | 11 | 19 | 86% |
| DRDS 0.9375 cps | 0.00011 | 0.00320 | 10.7 | 7.3 | 33 | 21 | 0 | 5 | 5 | 24% |
| DRDS 1.875 cps | 0.00000 | 0.00027 | 15.3 | 14.5 | 30 | 22 | 0 | 16 | 16 | 73% |
| DRDS 3.75 cps | 0.00012 | 0.00093 | 11.1 | 16.1 | 30.5 | 22 | 3 | 14 | 14 | 64% |

DRDC dynamic random dot correlograms, DRDS dynamic random dot stereograms, f1 fundamental frequency, f2 the first harmonic frequency, cps cycles per secundum, No. Number of, rec. recording, IQR interquartile range

**Supplementary Movies**

Two videos used for stereo stimulation in the study are attached to the article. Both videos run at 15 fps with a resolution of 640×480 px. The stimulus frequency is 1.875 cps. Each video contains 10 cycles, resulting in a total duration of 5.3 seconds at the given stimulus frequency. The colors used in the video, when viewed through TNO anaglyph glasses, produce the contrast and luminance values described in the article (see Methods).

- In DRDCVideo.avi, the video begins with 4 frames of the correlated phase, followed by 4 frames of the anticorrelated phase.

In DRDSVideo.avi, the first 4 frames display a checkerboard pattern with a 48 × 48 dot check size. Every other checkerboard pattern exhibits a crossed disparity of 2 dots, while the remaining ones exhibit an uncrossed disparity of 2 dots. Given the monitor size and viewing distance, this configuration resulted in ±7’ disparity in our setup.**Supplementary Figures**

Fig. S1 Summary of the participants’ responses evoked by the 0.9375 cps DRDC visual stimulus. Each recording is represented by a head model. The area of the green dots is proportional to the T^2^_circ_ value for the given recording at specific electrode positions, if the test result for that channel was significant. The dot representing the highest T^2^_circ_ value of the participant has the same area on each head model. The labels f1 or f2 indicate the frequency at which more of the channels showed significant ssVEP in the given recording. Red circles show electrode positions that were not significant in the given recording, while red x marks indicate electrodes that delivered noisy signal in the given recording identified by visual inspection (see Methods). In some cases, significant responses were detected even on such noisy channels.

Fig. S2 In the above image, we can see data related to the responses evoked by a 3.75 cps DRDC visual stimulus. Each recording is represented by a head model. The area of the green dots is proportional to the T^2^_circ_ value for the given recording at specific electrode positions, if the test result for that channel was significant. The dot representing the highest T^2^_circ_ value of the participant has the same area on each head model. The labels f1 or f2 indicate the frequency at which more of the channels showed significant ssVEP in the given recording. Red circles show electrode positions that were not significant in the given recording, while red x marks indicate electrodes that delivered noisy signal in the given recording identified by visual inspection (see Methods). In some cases, significant responses were detected even on such noisy channels.

Fig. S3 In the above image, we can see data related to the responses evoked by a 0.9375 cps DRDS visual stimulus. Each recording is represented by a head model. The area of the green dots is proportional to the T^2^_circ_ value for the given recording at specific electrode positions, if the test result for that channel was significant. The dot representing the highest T^2^_circ_ value of the participant has the same area on each head model. The labels f1 or f2 indicate the frequency at which more of the channels showed significant ssVEP in the given recording. Red circles show electrode positions that were not significant in the given recording, while red x marks indicate electrodes that delivered noisy signal in the given recording identified by visual inspection (see Methods). In some cases, significant responses were detected even on such noisy channels.

Fig. S4 In the above image, we can see data related to the responses evoked by a 1.875 cps DRDS visual stimulus. Each recording is represented by a head model. The area of the green dots is proportional to the T^2^_circ_ value of the T^2^_circ_ statistic for the given recording at specific electrode positions, if the statistic for that channel was significant. The dot representing the highest T^2^_circ_ value of the participant has the same area on each head model. The labels f1 or f2 indicate the frequency at which more of the channels showed significant ssVEP in the given recording. Red circles show electrode positions that were not significant in the given recording, while red x marks indicate electrodes that delivered noisy signal in the given recording identified by visual inspection (see Methods). In some cases, significant responses were detected even on such noisy channels.

Fig. S5 In the above image, we can see data related to the responses evoked by a 3.75 cps DRDS visual stimulus. Each recording is represented by a head model. The area of the green dots is proportional to the T^2^_circ_ value for the given recording at specific electrode positions, if the test result for that channel was significant. The dot representing the highest T^2^_circ_ value of the participant has the same area on each head model. The labels f1 or f2 indicate the frequency at which more of the channels showed significant ssVEP in the given recording. Red circles show electrode positions that were not significant in the given recording, while red x marks indicate electrodes that delivered noisy signal in the given recording identified by visual inspection (see Methods). In some cases, significant responses were detected even on such noisy channels.
